# Supplementary material for: “What Do We Know about Hope in Nursing Care?”: A Synthesis of Concept Analysis Studies
Source: Healthcare (Basel). 2023 Oct 14;11(20):2739. doi: 10.3390/healthcare11202739 (PMC10606526; doi:10.3390/healthcare11202739)
Supplement: Supplementary file 1 [file healthcare-11-02739-s001.zip › healthcare-2562527-supplementary.pdf]

Search Protocol

Search engine: PubMed

Search	*Search Equation*	*Results*
#1	hope	719
#2	concept	2,525
#3	analysis	101,044
#4	nur*	423
#5	1 AND 2 AND 3 AND 4	204

Search engine: Cinahl

Search	*Search Equation*	*Results*
#1		9,269
#2		21,752
#3		271,192
#4		393,752
#5		27

Search engine: Medline

Search	*Search Equation*	*Results*
#1		12,737
#2		81,510
#3		1,230,848
#4		296,659
#5		39

Search engine: Scopus

Search	*Search Equation*	*Results*
#1		126,064

#2		55,468
#3		1,829
#4		1,540
#5		50
